# Correction: Impaired speed encoding and grid cell periodicity in a mouse model of tauopathy

**DOI:** 10.7554/eLife.87504

**Published:** 2023-03-07

**Authors:** Thomas Ridler, Jonathan Witton, Keith G Phillips, Andrew Randall, Jonathan T Brown

**Keywords:** Mouse

Ridler T, Witton J, Phillips KG, Randall AD, Brown JT. 2020. Impaired speed encoding and grid cell periodicity in a mouse model of tauopathy. *eLife* **9**:e59045. doi: 10.7554/eLife.59045.

Published 26 November 2020

In Figure 2—figure supplement 1A, two of the panels (bottom row, column 3 and 4) contained images showing the wrong portion of the histological sections, highlighting the incorrect lesion sites. The figure has been remade with images from the correct area. Additionally, for each of the sections, an image has been included at lower magnification, so that the viewer can see the brain regions and surrounding landmarks more clearly. We have included the source images for these images in Figure 2—figure supplement 1-source data 1.

Since it is possible that variations in electrode placement may account some of our experimental results, we have also added an addition paragraph for the discussion to account for this.

Original figure:

**Figure 1:**
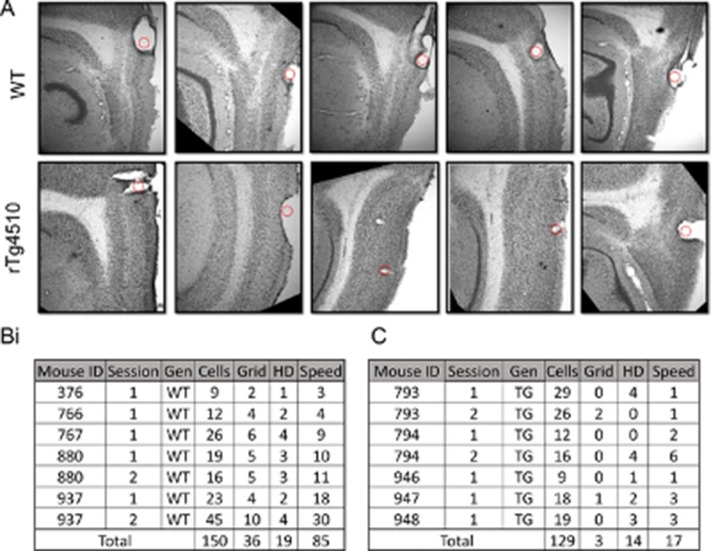


Corrected Figure:

**Figure 2:**
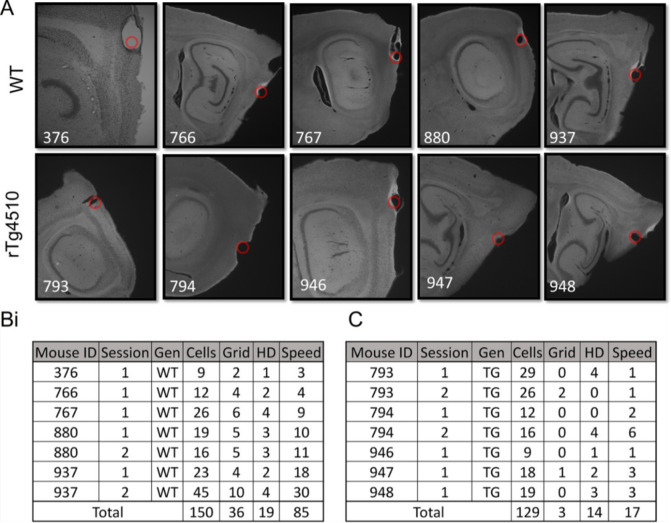


**Additional source data**

Figure 2—figure supplement 1—source data 1.: Example images of electrolytic lesions for each mouse.

**Additional discussion**

While we are confident that we recorded the activity of MEC single units, it is also possible that variations in electrode placements across genotypes could have influenced our results. Incorrectly placed electrodes alone could account for a reduction in grid cells in the rTg4510 group. The pronounced cortical shrinkage produced by this model makes the precise positioning of electrode arrays difficult to achieve using standard stereotaxic coordinates.

## Additional files

Figure 2—figure supplement 1—source data 1. Example images of electrolytic lesions for each mouse.

