# Supplementary figures and images for: Impaired speed encoding and grid cell periodicity in a mouse model of tauopathy
Source: eLife. 2020 Nov 26;9:e59045. doi: 10.7554/eLife.59045 (PMC7690954; doi:10.7554/eLife.59045)

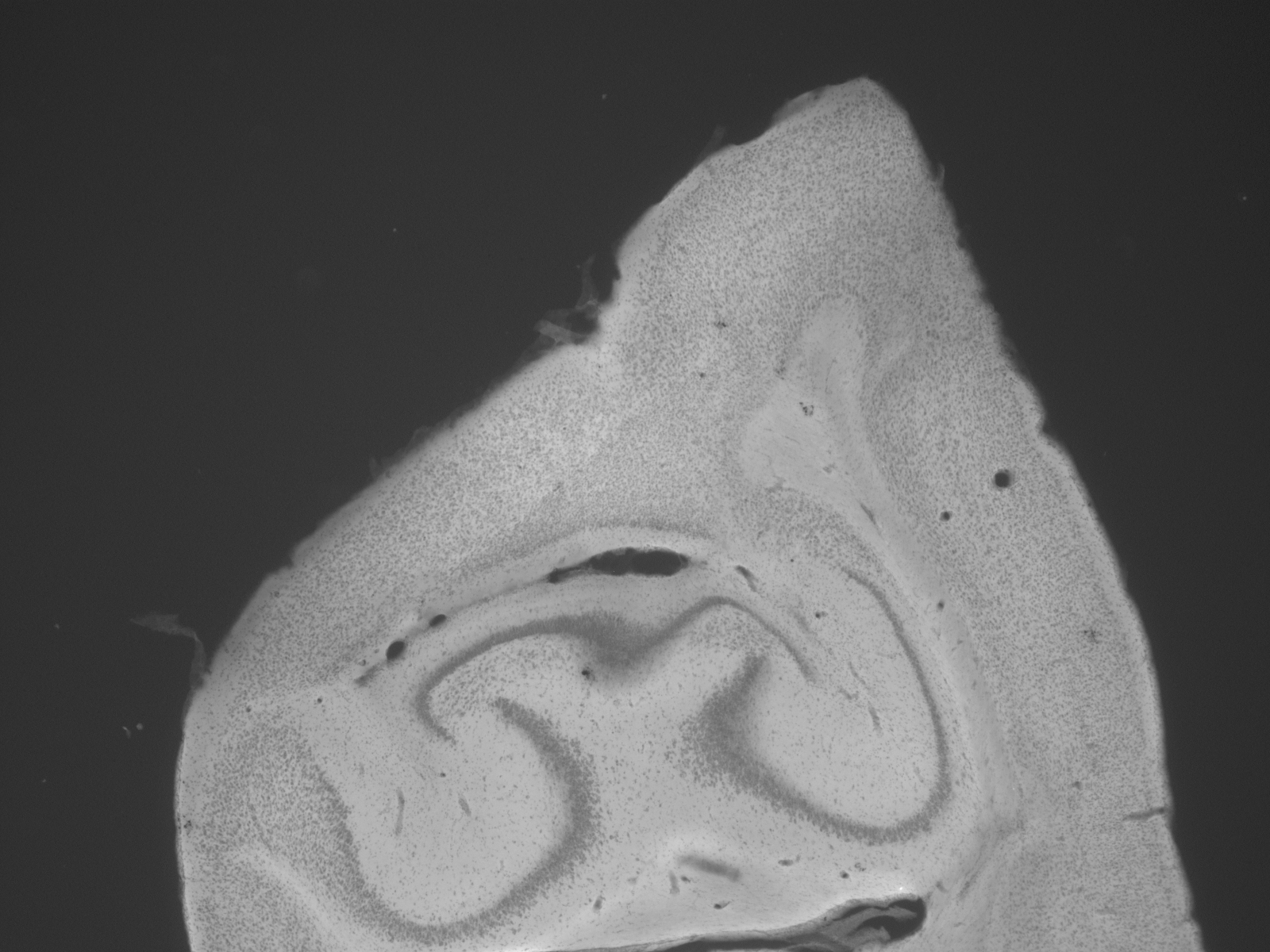

Supplement: Figure 2—figure supplement 1—source data 1. [file elife-59045-fig2-figsupp1-data1.zip › Figure 2ΓÇöfigure supplement 1-Source data 1 /rTg4510/947.tif]

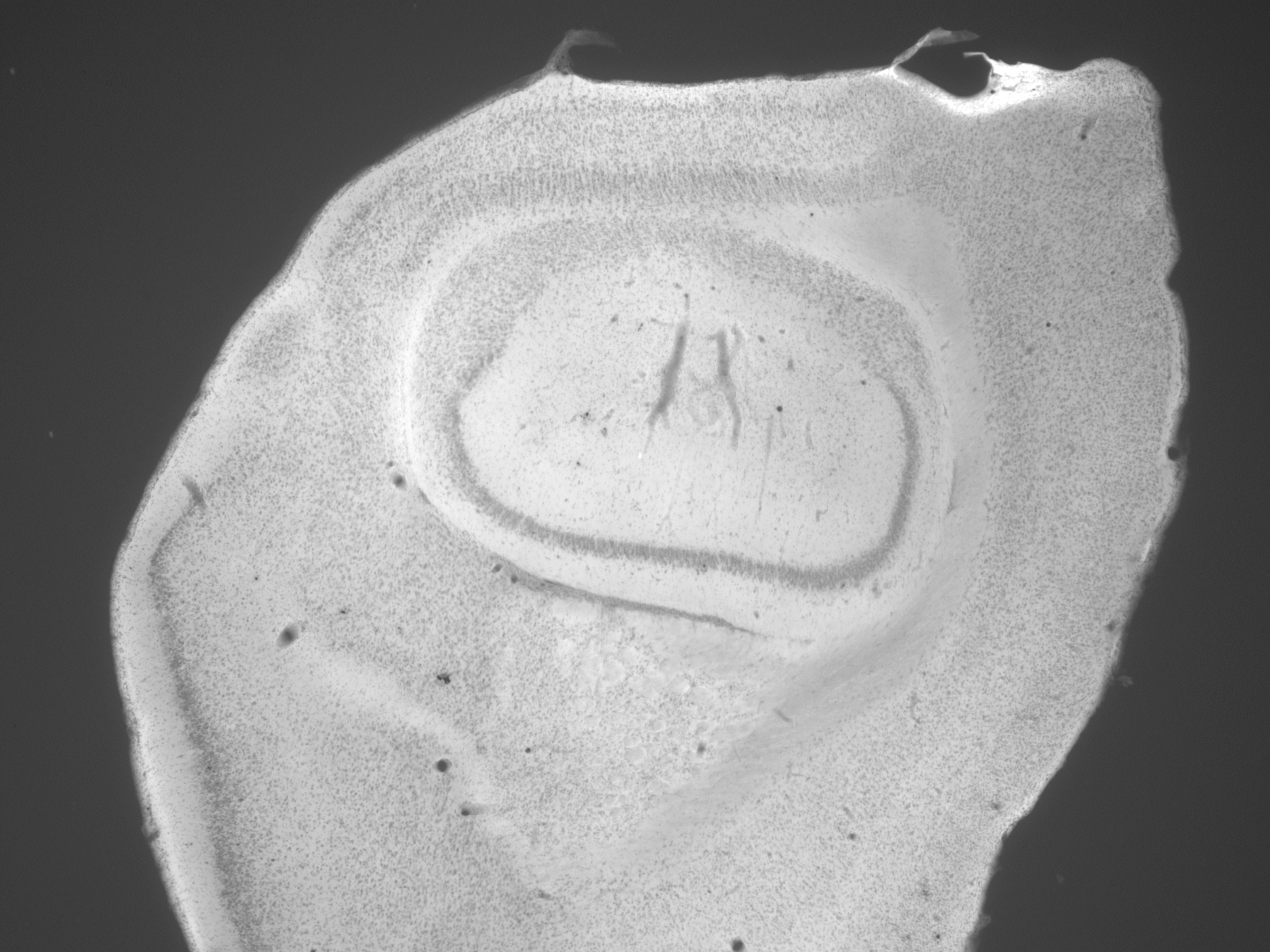

Supplement: Figure 2—figure supplement 1—source data 1. [file elife-59045-fig2-figsupp1-data1.zip › Figure 2ΓÇöfigure supplement 1-Source data 1 /rTg4510/946.tif]

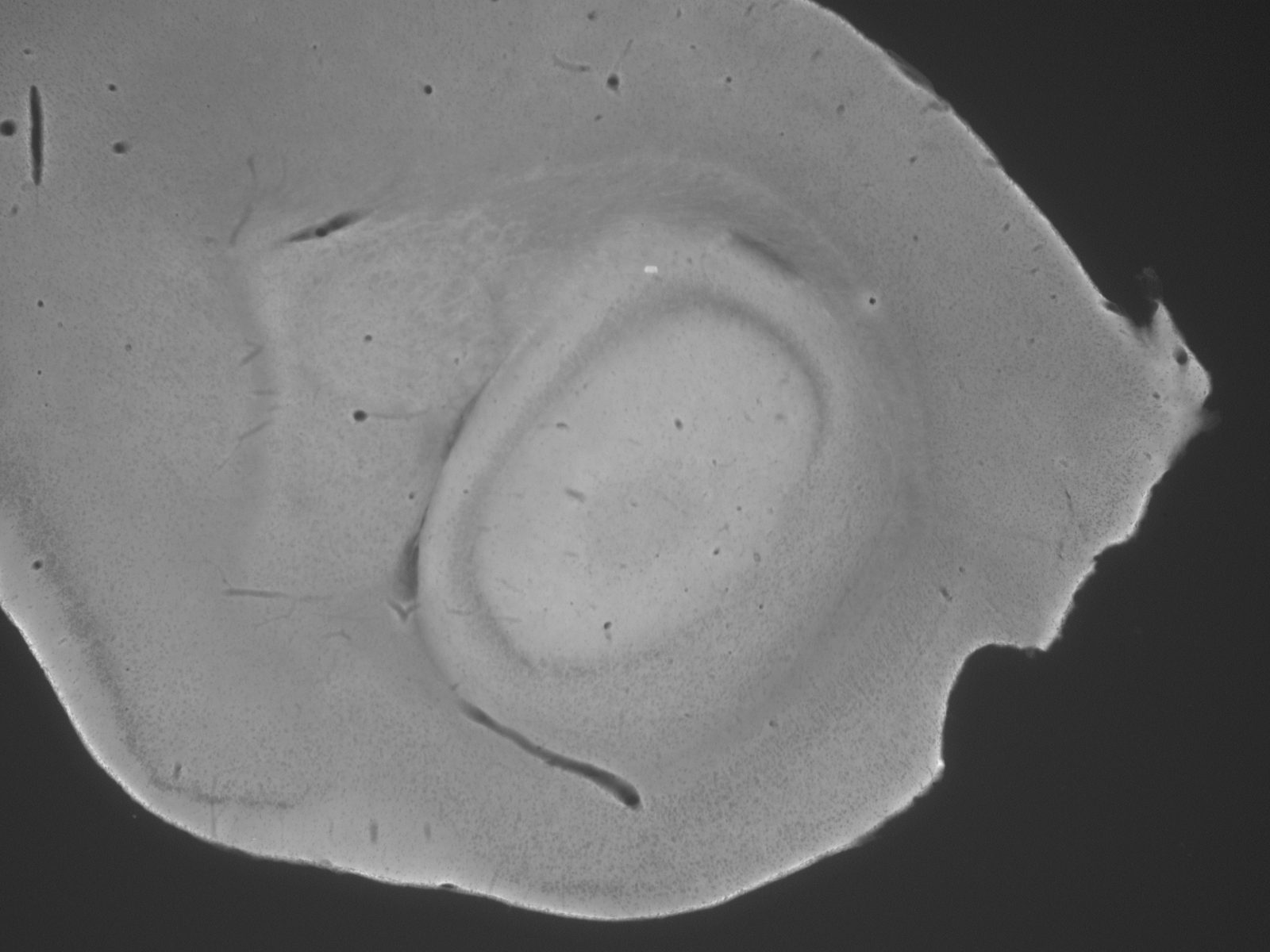

Supplement: Figure 2—figure supplement 1—source data 1. [file elife-59045-fig2-figsupp1-data1.zip › Figure 2ΓÇöfigure supplement 1-Source data 1 /rTg4510/794.tif]

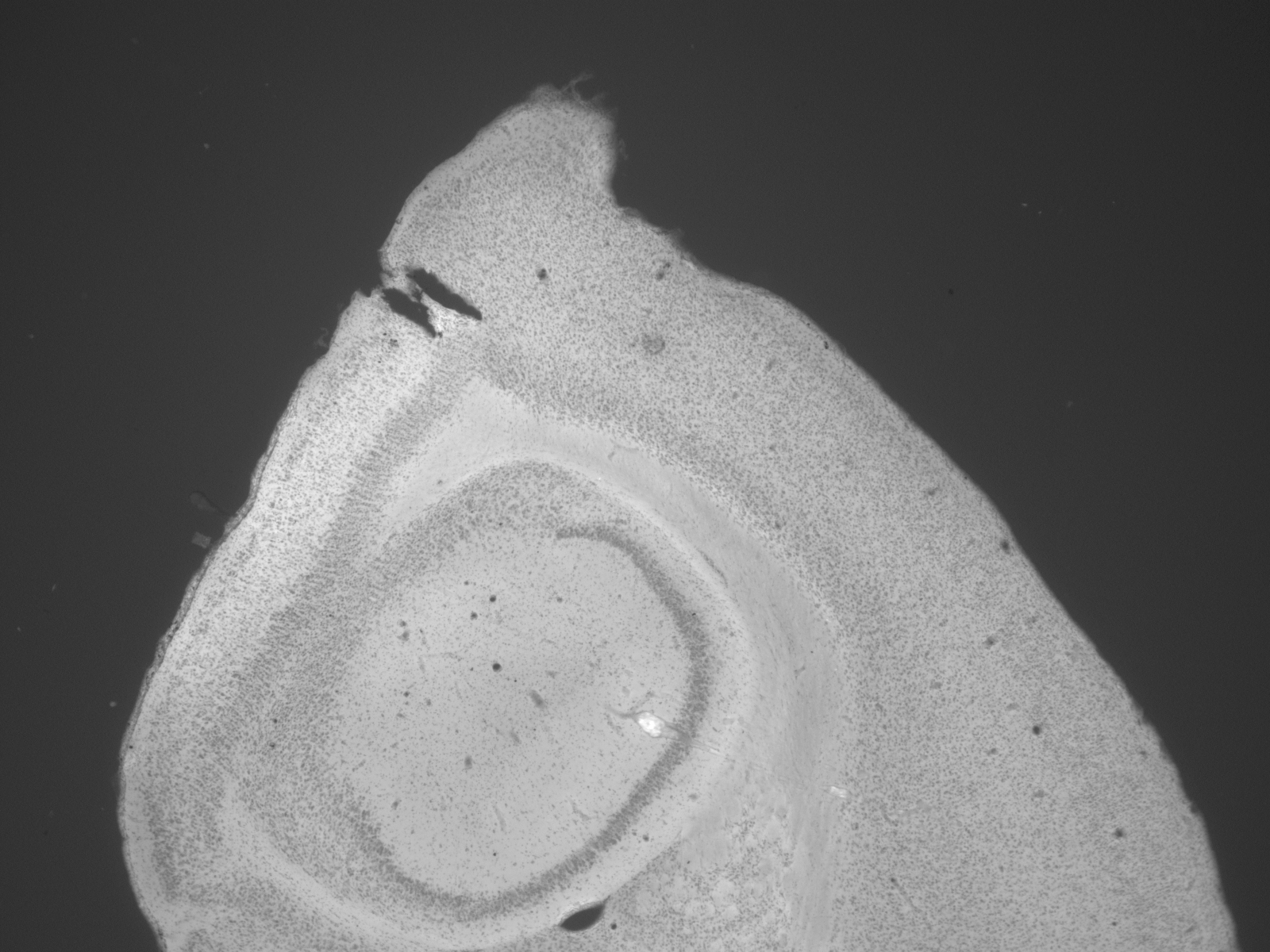

Supplement: Figure 2—figure supplement 1—source data 1. [file elife-59045-fig2-figsupp1-data1.zip › Figure 2ΓÇöfigure supplement 1-Source data 1 /rTg4510/793.tif]

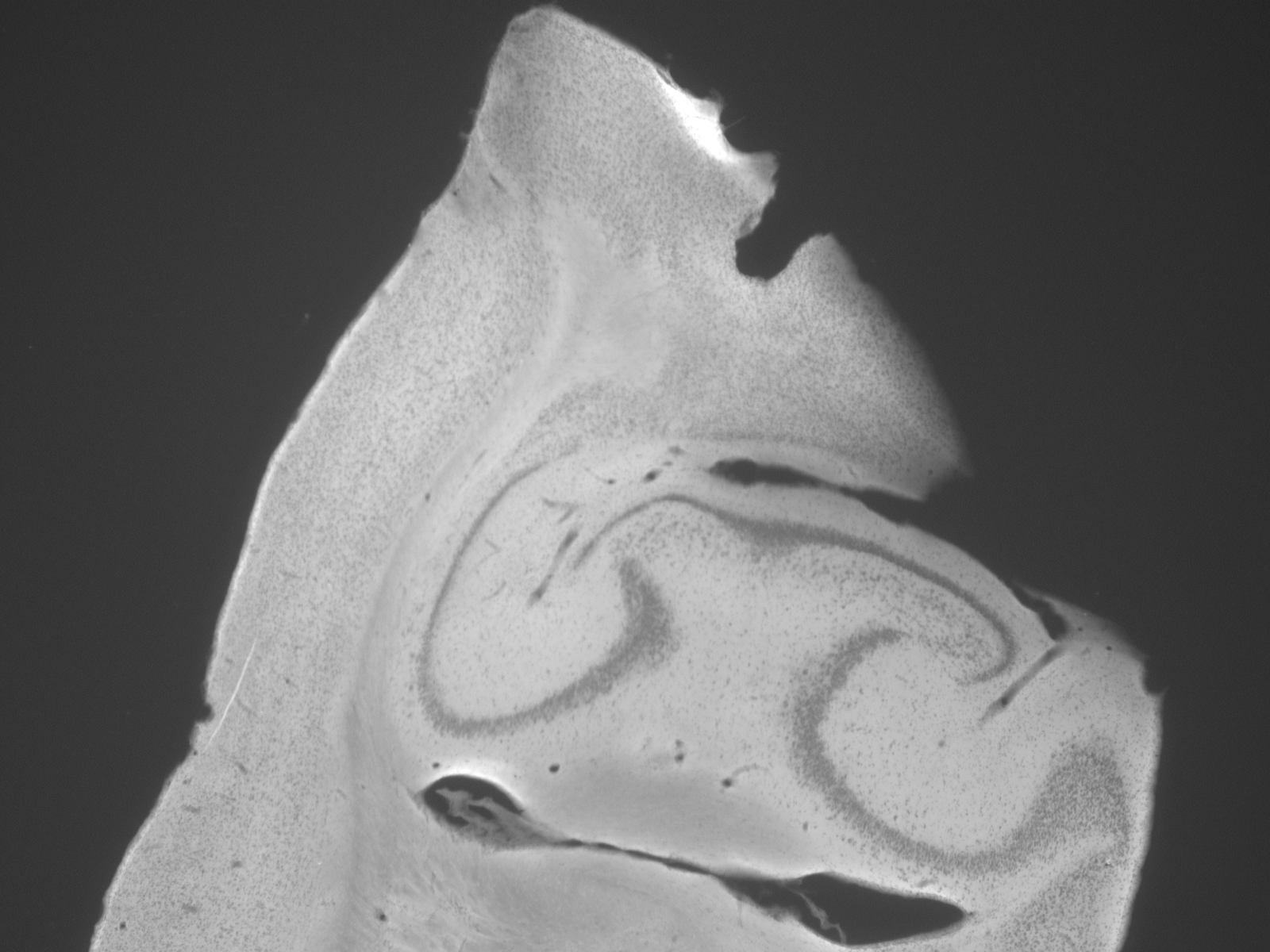

Supplement: Figure 2—figure supplement 1—source data 1. [file elife-59045-fig2-figsupp1-data1.zip › Figure 2ΓÇöfigure supplement 1-Source data 1 /rTg4510/948.tif]

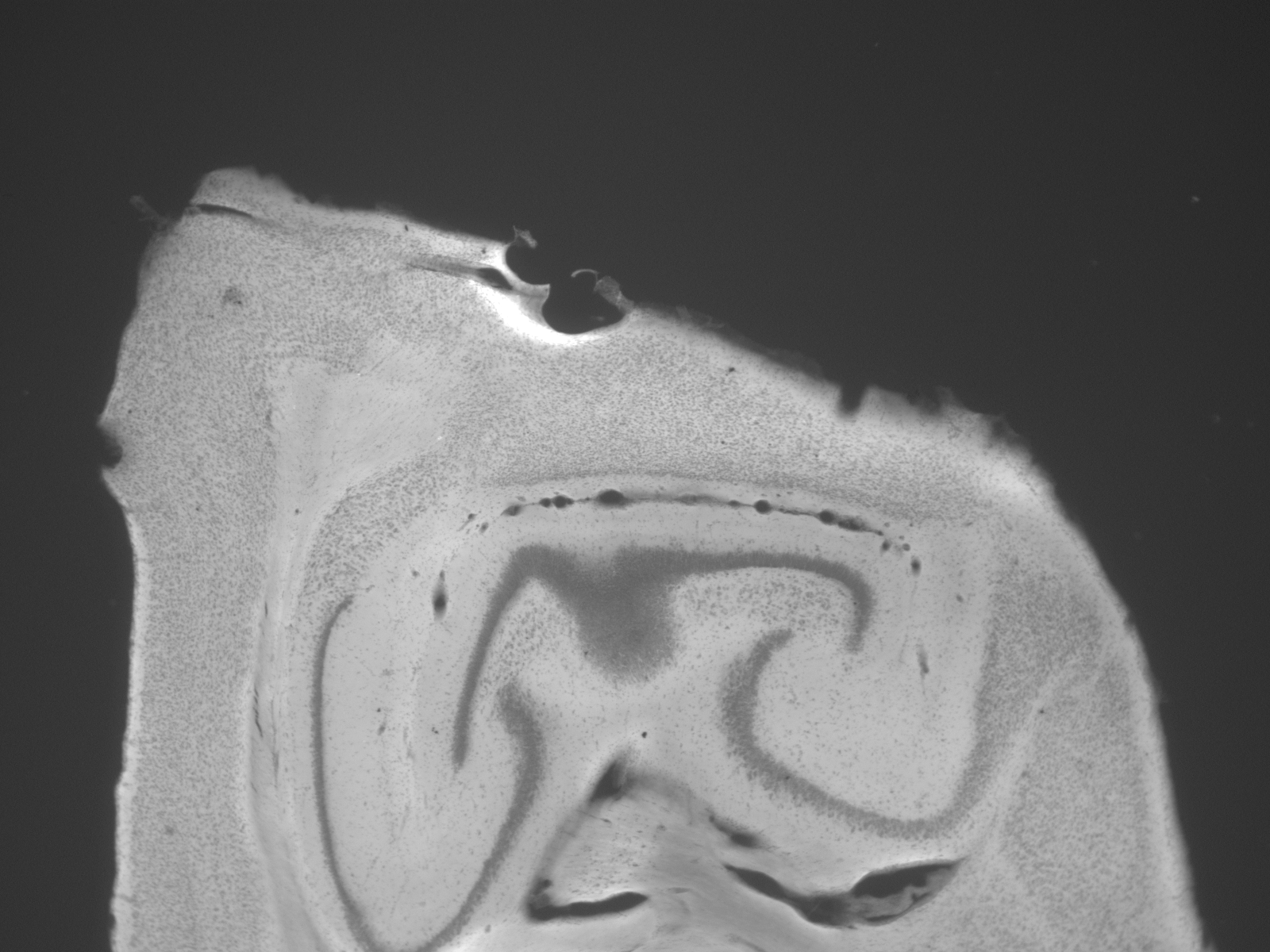

Supplement: Figure 2—figure supplement 1—source data 1. [file elife-59045-fig2-figsupp1-data1.zip › Figure 2ΓÇöfigure supplement 1-Source data 1 /WT/937.tif]

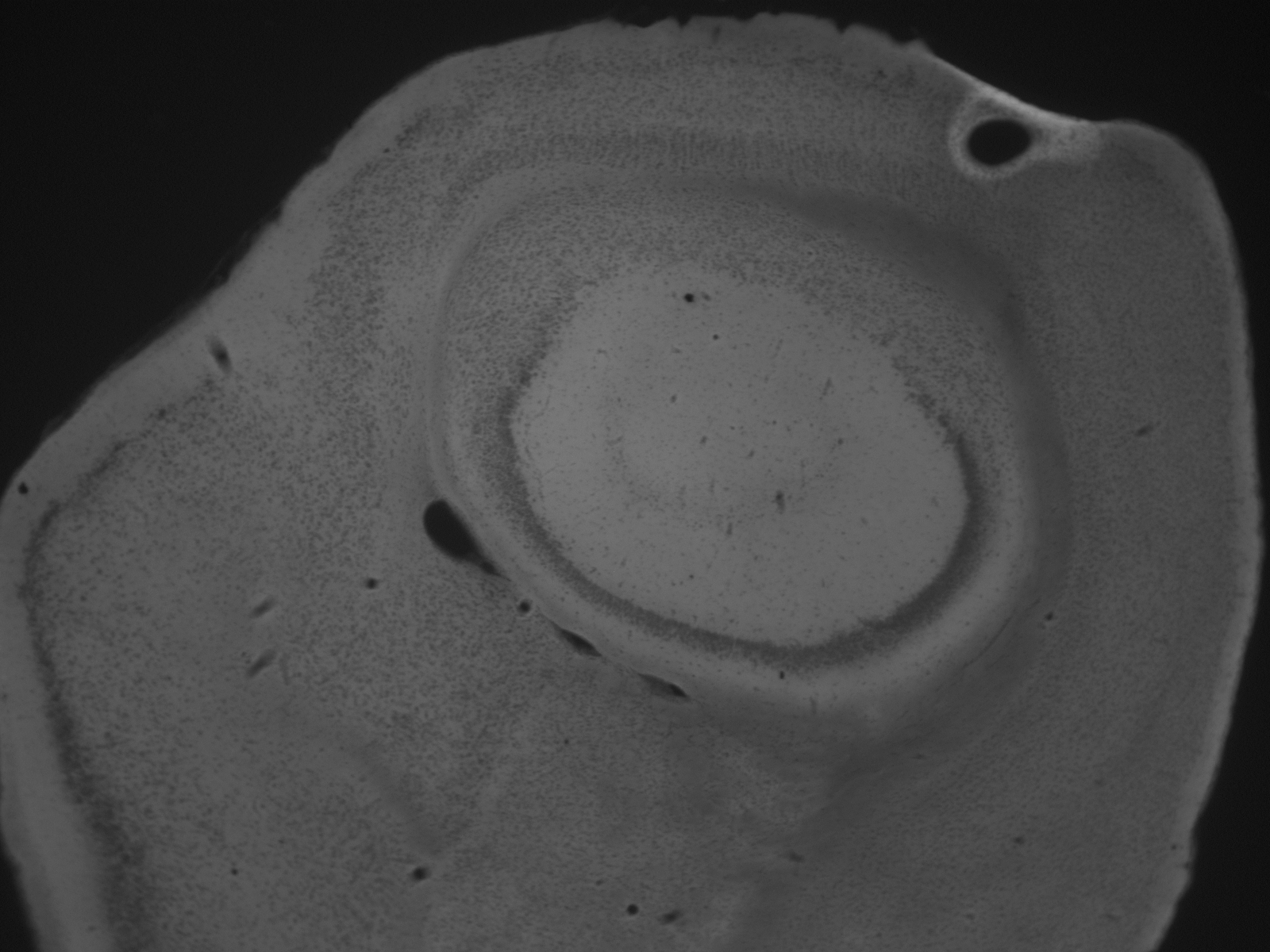

Supplement: Figure 2—figure supplement 1—source data 1. [file elife-59045-fig2-figsupp1-data1.zip › Figure 2ΓÇöfigure supplement 1-Source data 1 /WT/880.tif]

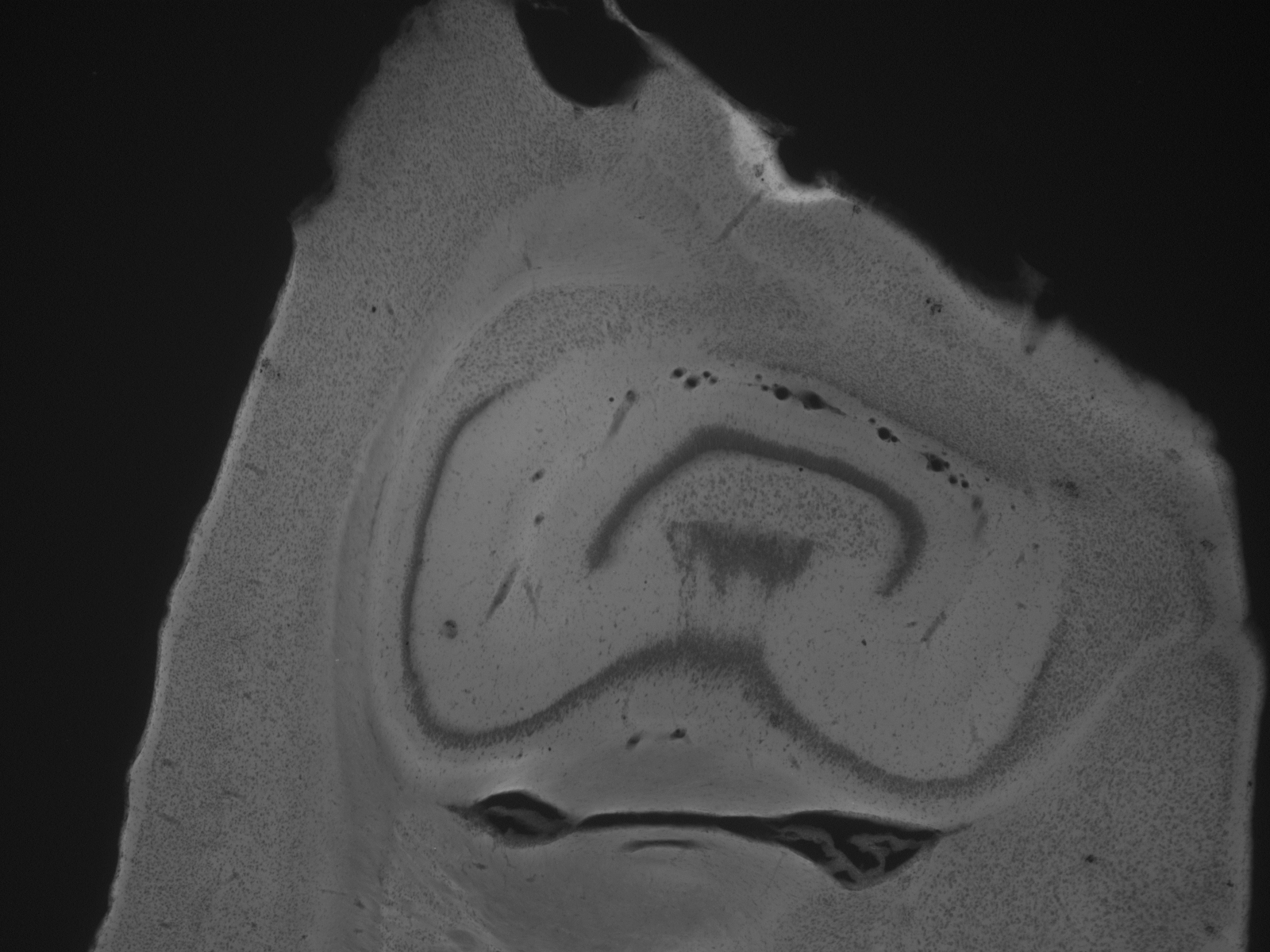

Supplement: Figure 2—figure supplement 1—source data 1. [file elife-59045-fig2-figsupp1-data1.zip › Figure 2ΓÇöfigure supplement 1-Source data 1 /WT/766.tif]

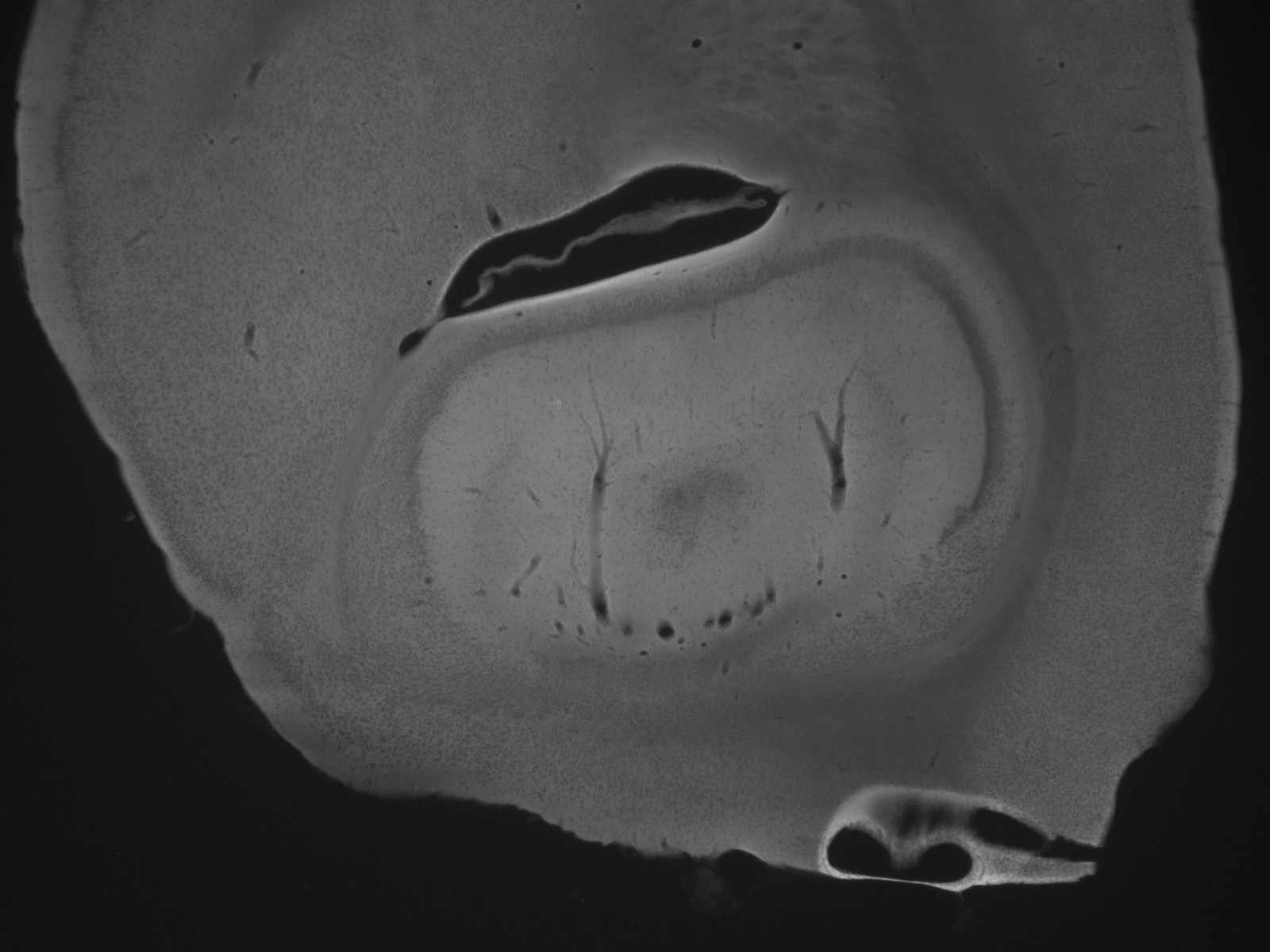

Supplement: Figure 2—figure supplement 1—source data 1. [file elife-59045-fig2-figsupp1-data1.zip › Figure 2ΓÇöfigure supplement 1-Source data 1 /WT/767.tif]
